# Corneal pannus, Herbert’s pits and conjunctival inflammation in older children in Papua New Guinea

**DOI:** 10.1080/09286586.2023.2273507

**Published:** 2024-02-08

**Authors:** Gillian M. Cochrane, Magdelene Mangot, Wendy Houinei, Melinda Susapu, Anasaini Cama, Richard Le Mesurier, Sara Webster, Tessa Hillgrove, Jaki Barton, Robert Butcher, Emma M. Harding-Esch, David Mabey, Ana Bakhtiari, Andreas Müller, Aya Yajima, Anthony W. Solomon, John Kaldor, Samuel Peter Koim, Robert Ko, Jambi Garap

**Affiliations:** a Collaborative Vision, Melbourne, Australia; b National Prevention of Blindness Committee, Port Moresby, Papua New Guinea; c National Department of Health, Port Moresby, Papua New Guinea; d Fred Hollows Foundation, Sydney, Australia; e Clinical Research Department, London School of Hygiene & Tropical Medicine, UK; f International Trachoma Initiative, Task Force for Global Health, Atlanta, USA; g Department of Noncommunicable Diseases, World Health Organization, Geneva, Switzerland; h Division of Programmes for Disease Control, Western Pacific Regional Office, World Health Organization, Manila, Philippines; i Global Neglected Tropical Diseases Programme, World Health Organization, Geneva, Switzerland; j Kirby Institute, University of New South Wales, Sydney, Australia; k PNG Eye Care, Port Moresby, Papua New Guinea

**Keywords:** Trachoma, corneal limbus, pannus, Herbert’s pits, Papua New Guinea

## Abstract

**Purpose:**

The prevalence of trachomatous inflammation—follicular (TF) in Papua New Guinea (PNG) suggests antibiotic mass drug administration (MDA) is needed to eliminate trachoma as a public health problem but the burden of trichiasis is low. As a result, WHO issued bespoke recommendations for the region. If ≥ 20% of 10–14-year-olds have both any conjunctival scarring (C1 or C2 or C3) and corneal pannus and/or Herbert’s pits, MDA should be continued. Equally, if ≥ 5% of that group have both moderate/severe conjunctival scarring (C2 or C3) and corneal pannus and/or Herbert’s pits, MDA should be continued.

**Methods:**

We identified 14 villages where > 20% of 1–9-year-olds had TF during baseline mapping undertaken 4 years and 1 month previously. Every child aged 10–14 years in those villages was eligible to be examined for clinical signs of corneal pannus, Herbert’s pits and conjunctival scarring. A grading system that built on existing WHO grading systems was used.

**Results:**

Of 1,293 resident children, 1,181 (91%) were examined. Of 1,178 with complete examination data, only one (0.08%) individual had concurrent scarring and limbal signs.

**Conclusions:**

The WHO-predefined criteria for continuation of MDA were not met. Ongoing behavioural and environmental improvement aspects of the SAFE strategy may contribute to integrated NTD control. Surveillance methods should be strengthened to enable PNG health authorities to identify future changes in disease prevalence.

## Introduction

Trachoma is the leading infectious cause of blindness worldwide and in 2021 was known to be a public health problem in 44 countries.^1,2^ The pathogenesis of blindness from trachoma occurs over many years. Inflammation caused by repeated infection with ocular *Chlamydia trachomatis (Ct)* in childhood can lead to conjunctival scarring.^3,4^ In severe cases, the eyelids become deformed causing eyelashes to abrade the cornea (trichiasis) causing pain and corneal opacity that can lead to blindness.^5–7^ Trachoma is considered a public health problem in areas where (a) the prevalence of trachomatous inflammation—follicular (TF) in 1–9-year-olds is ≥ 5% and/or (b) the prevalence of trachomatous trichiasis (TT) in ≥ 15-year-olds is ≥ 0.2%.^8^

To reduce the risk of progression to blindness from trachoma, the World Health Organization (WHO) recommends implementation of the SAFE strategy (surgery, antibiotics, facial cleanliness, environmental improvement) at the district level. The antibiotic component is delivered as annual mass drug administration (MDA) of oral azithromycin (or tetracycline eye ointment in those in whom azithromycin is contraindicated), with the number of years of delivery depending on the prevalence of TF in 1–9-year-olds. Conducting MDA using a broad-spectrum antibiotic such as azithromycin carries a risk of selection of antibiotic resistance in bacteria in the treated area.^9–12^ There are also considerable financial and logistic challenges to conducting MDA, especially in isolated or difficult-to-access populations.^13–15^ For those reasons, decisions to undertake MDA should offer defined benefit to the populations treated.

In Papua New Guinea (PNG) and other parts of Melanesia, TT prevalence is very low despite an appreciable burden of TF.^16–19^ The prevalence and estimated transmission intensity of ocular *Ct* and the prevalence of conjunctival scarring are also low.^20–23^ A number of explanations could plausibly account for this pattern, although no evidence has been uncovered to support any specific theory.^19,24–26^ The combination of moderate-to-high TF prevalence and very low TT prevalence has triggered significant discussion at the regional and global level about whether MDA for trachoma should be delivered in these settings.^26,27^ As a result of these discussions, TF prevalence was not felt to be a valid marker of the need for intervention in this setting and a novel survey strategy was developed to determine whether children living in previously high-TF communities had other, longer-lived clinical signs of trachoma.^26,28^ Specifically, the corneal signs of pannus and Herbert’s pits (HPs; collectively termed limbal signs in this manuscript) are thought to be highly specific for previous active trachoma.^29,30^ The core tenet of this novel survey design is that, when found in conjunction with limbal signs, conjunctival scarring is due to trachoma and that individuals with that combination are at risk of future blindness from trachoma. WHO recommended that, where 20% of 10–14-year-olds have any degree of scarring (C1 or C2 of C3, according to the 1981 WHO trachoma grading scheme,^31^ plus limbal signs in the worst affected eye, or where 5% have moderate-to-severe scarring (C2 or C3), plus limbal signs, MDA would be justified to reduce a high prevalence of TF in children.^26^

The purpose of this study was to determine the prevalence of limbal signs and conjunctival scarring in 10–14-year-olds in previously high-TF communities in PNG.

## Methods

### Study ethics

The study adhered to the tenets of the Declaration of Helsinki and was approved by the PNG Medical Research Advisory Committee (19.09).

### Study rationale and outcome measure

This survey methodology was designed to recruit older children who were known to have grown up in high-TF environments in Melanesia.^26,28^ This methodology was designed for rapid deployment, thus focused on communities with the highest levels of TF in children during pre-MDA trachoma prevalence surveys.^16^

As this was a novel survey methodology looking for signs not routinely recorded under the commonly used WHO simplified trachoma grading system, a grading scheme (Table 1) was assembled from existing WHO protocols and assessment systems^31–33^ with minimal modification. Grading using this combination of schemes has previously been used in the Solomon Islands and Vanuatu.^28^

### Study population

**Figure 1:**
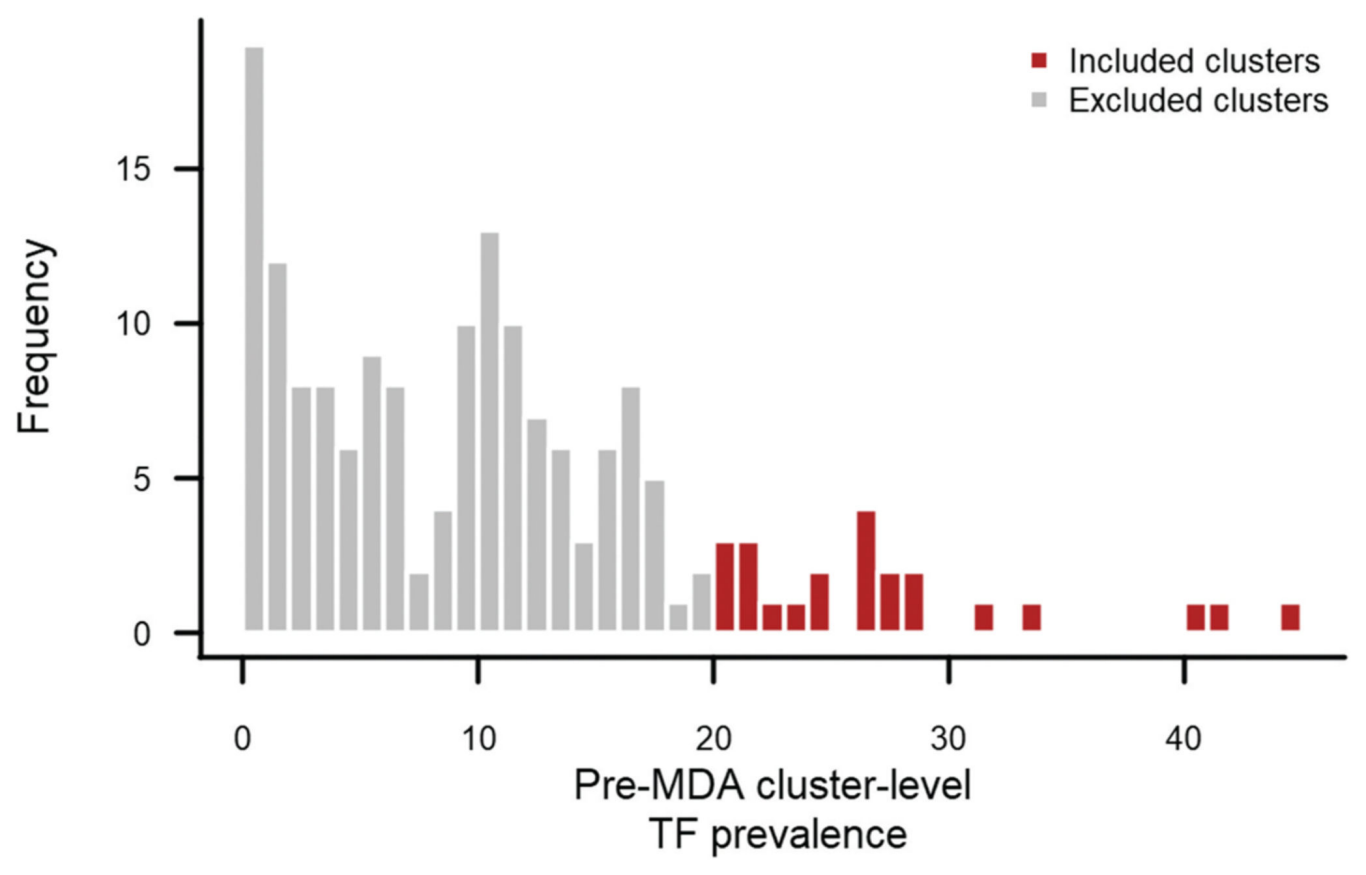
Age- and gender-adjusted cluster-level TF prevalence in children aged 1–9 years in Papua New Guinea from baseline surveys conducted with the Global Trachoma Mapping Project in December 2015.^16^

We wanted to recruit participants from communities previously known to have high proportions of children with TF. Therefore, high-TF clusters were selected from the pre-MDA trachoma survey.^16^ Age- and gender-adjusted cluster-level prevalence of children aged 1–9 years with TF in all six mapped evaluations units was calculated from PNG’s Global Trachoma Mapping Project dataset (Figure 1). Clusters with an adjusted TF prevalence ≥20% were selected for inclusion.

### Implementation

Teams set out to register all 10–14-year-olds resident in selected communities: individuals who would have been ~ 5–9-year-olds at the time of the 2015 baseline surveys. It was assumed *a priori* that there was limited population mobility amongst this age group due to their age. Verbal approval to enrol villages in the study was sought from village leaders. Participants were recruited by teams going house-to-house. Household heads were asked how many 10–14-year-olds lived in the house. If a child in that age range was absent (for example, at school), a return visit to the household was made. Individuals were provided with information about the study and asked to provide verbal assent to take part. As all participants were aged <18 years, where assent was given, a parent or guardian was required to provide written, informed consent.

One ophthalmologist was assigned to each of four teams. The ophthalmologists were trained by an experienced ophthalmologist (RLM) in the grading of conjunctival and corneal limbus features using photographs. Graders practiced examination of the conjunctiva and sclero-corneal junction on live subjects without limbal signs. Each individual enrolled was assessed for conjunctival scarring, upper pole corneal pannus and Herbert’s pits, against the criteria in Table 1. Children with suspected active trachoma were treated with tetracycline eye ointment, provided free of charge, and anyone found with TT was referred to local ophthalmic services for appropriate management.

Data were collected using smartphone-based electronic data collection forms, encrypted and uploaded to a secure Cloud-based server on completion. The recorder was trained in the use of the smartphone and familiarised with data collection forms. Cloud servers were hosted by Tropical Data, and a copy of the raw data made available to all study co-investigators via a secure link once data collection was confirmed to have been completed. Duplicate records were defined as individuals with the same recorded name, age and gender in the same household. These records (three duplicate pairs) were removed from the dataset prior to analysis.

The primary outcomes were the prevalence of defined combinations of signs in 10–14-year-olds, as defined at WHO’s 2018 *Expert Consultation on Trachoma in the Pacific Islands*,^26^ specifically (i) limbal signs (pannus, HPs or both) in at least one eye plus conjunctival scarring (C ≥ 1) in at least one eye; and (ii) limbal signs (pannus, HPs or both) in at least one eye and moderate-to-severe conjunctival scarring (C ≥ 2) in at least one eye. An individual was determined to have limbal disease if they had upper pole corneal pannus ≥2 mm and/or at least one HP in at least one eye. An individual was determined to have conjunctival scarring if at least one eye had a scar grade of C1 or more. The outcome of the study was determined at the individual level (i.e., limbal disease and scarring did not need to be in the same eye for an individual to be considered a case). Where disease grades are presented, they represent the individual’s most severely affected eye.

## Results

Of 169 clusters surveyed under baseline mapping, 23 had a TF prevalence ≥ 20%. Of these, 14 clusters were included in this survey. Nine clusters were excluded: four clusters in Madang province because of security concerns; three clusters in Morobe province as baseline data were not completed or published; and two clusters from West New Britain province because their cluster identification numbers could no longer be linked to village names or locations.

### Population studied

Data collection took place over two weeks in January 2020. From the 744 surveyed households in the 14 study villages, 1293 children aged 10–14 years were resident, of whom 1181 (91%) were examined. Of the 112 who were not examined, 108/112 (96%) were absent and 4/112 (4%) refused. The median number of children examined per village was 82 (range: 31–168).

### Clinical examination

**Figure 2:**
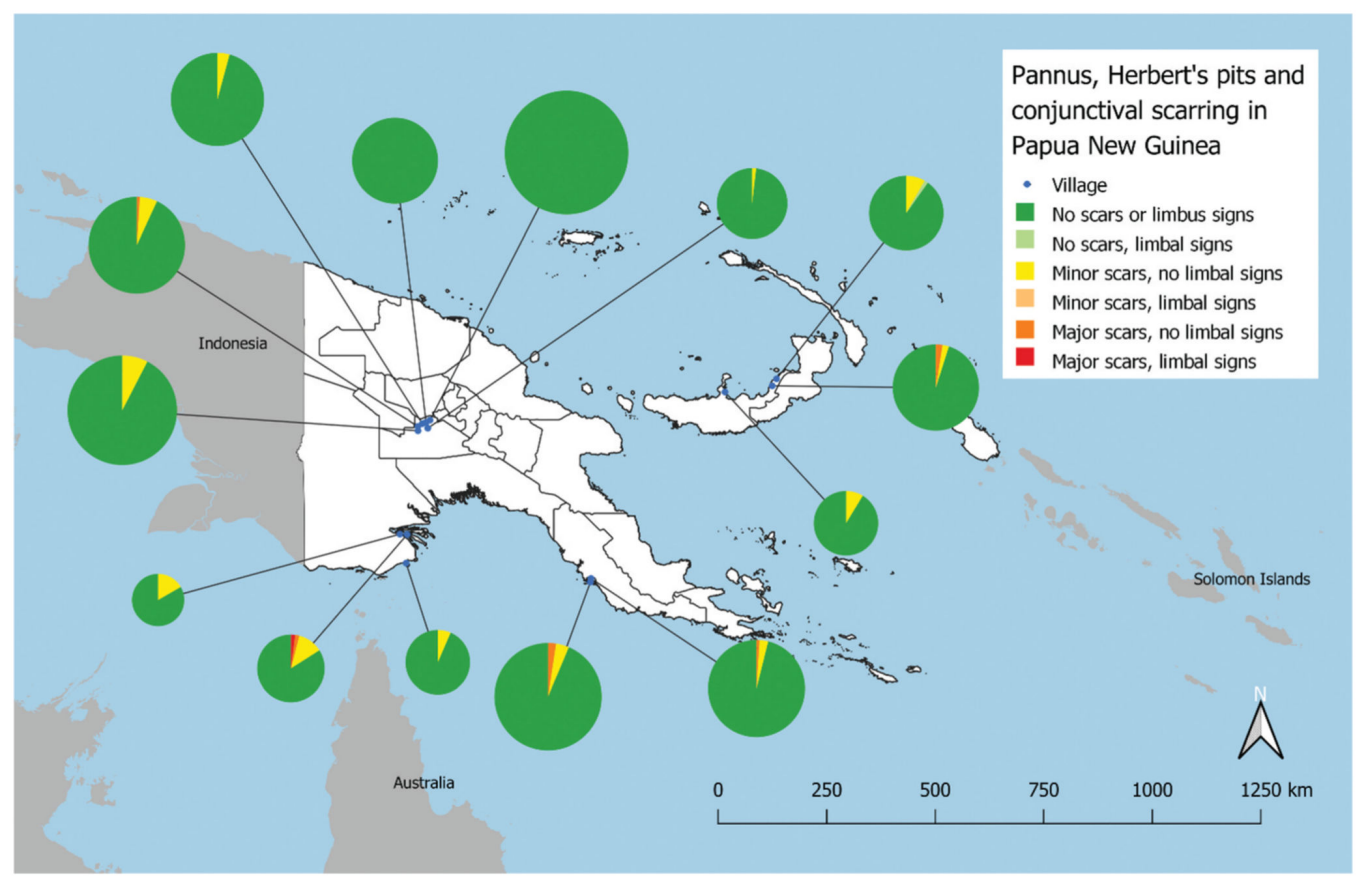
Geographical distribution of clinical signs of trachoma in 14 selected villages of Papua New Guinea, 2020. ‘Minor scars’ refers to an individual with conjunctival scarring graded C1 in at least one eye. ‘Major scars’ refers to an individual with conjunctival scarring graded C2 or C3 in at least one eye. ‘Limbal signs’ refers to an individual with pannus ≥2 mm and/or ≥1 Herbert’s pit in at least one eye. The severity is based on the most severely affected eye in an individual. The boundaries and names shown and the designations used on this map do not imply the expression of any opinion whatsoever on the part of the authors, or the institutions with which they are affiliated, concerning the legal status of any country, territory, city or area or of its authorities, or concerning the delimitation of its frontiers or boundaries.

Three individuals were removed from the dataset of 1181 children examined: one whose right limbus and conjunctiva could not be graded, one in whom HPs could not be graded and one in whom neither right nor left limbus and conjunctiva could be examined. The reasons for non-grading/examination were not recorded. Analysis was undertaken on complete data from 1178 children (Table 2). Some degree of scarring was present in 63/1178 (5%) of children examined, of whom 54/63 (86%) were graded as having C1 and 9/63 (14%) were graded as having C2. There were only two cases of limbal signs, one of pannus (unilateral) and one of Herbert’s pits (bilateral). These were not in the same individual. The geographical distribution of these signs is shown in Figure 2. There was 1/1178 (0.08%) individual with any scar (C1 or C2 or C3) and any limbal sign. There was also 1/1178 (0.08%) individual with a moderate-to-severe scar (C2 or C3) and any limbal sign (Table 3).

## Discussion

The prevalence of conjunctival scars and limbal signs of trachoma in this study were both low. Only one individual out of 1178 had both conjunctival scars and limbal signs, and so the predefined criteria for continuation of MDA were not met.^26^ This result is supported by previous data on TT prevalence, ocular *Ct* prevalence and transmission that suggest trachoma in PNG is less severe than in other parts of the world.^16,23^ Historic reports and clinic surveys have also suggested the sight-threatening late stages of trachoma are rare in PNG.^19^ Furthermore, primary data and systematic reviews of causes of blindness in the region suggest that trachoma makes a negligible contribution to vision impairment in PNG and elsewhere in the Pacific.^34–36^ There is therefore consistency in the epidemiological picture of trachoma as it relates to blindness in PNG and its neighbours.

Our survey methodology was conceived in response to an unusual clinical presentation of disease in the Pacific. Consequently, there are very few comparator data. In the Solomon Islands and Vanuatu, 7% had concurrent conjunctival scar (C1 or C2 or C3) and limbal signs and 2% had moderate-to-severe conjunctival scar and limbal signs.^28^ There, too, the predefined criteria for the continuation of MDA were not met. Vanuatu was subsequently validated by WHO as having eliminated trachoma as a public health problem in August 2022, with the limbal sign data included along-side serological data as part of the dossier.^8^

There are positive and negative implications of the cessation of MDA for trachoma elimination. First, PNG is a challenging environment in which considerable human and financial resources will be saved by not doing MDA. Second, the attributable risk of selecting antimicrobial resistance through MDA is removed. Conversely, delivery of MDA for trachoma may have offered off-target benefits (for example, in the treatment of sexually transmitted infections or yaws^37,38^ or decreasing all-cause child mortality).^39^ Furthermore, an active trachoma programme may have attracted increased advocacy and investment in water, sanitation and hygiene (WASH) service development. Nevertheless, the balance of evidence suggests that PNG does not currently warrant intensive interventions specifically directed against trachoma.

There were limitations to this study. First, the clinical signs examined for here are not commonly assessed and there is not extensive programmatic experience in training and standardising staff to grade them. Furthermore, they can be difficult to see without a slit lamp (in the case of old corneal pannus and HPs) and can be subjective (in the case of mild conjunctival scarring). Together these challenges make it difficult to reproducibly grade these signs at the programmatic level. This was demonstrated by poor agreement in photograding during previous studies in the Solomon Islands and Vanuatu.^28^ Second, while corneal pannus and HPs are assumed among ophthalmologists to be highly specific for trachoma, there are very few empirical data to support that firm linkage or demonstrate their relevance in predicting risk of future trachomatous blindness. There is some evidence that corneal pannus has a similar positive predictive value to TF in identifying ocular *Ct*,^40^ but no prospectively designed longitudinal data comparing progression rates among those with and without these limbal signs. Third, the sampling method was non-random and focussed on villages for which we had existing data, with an assumption that the children in the village were the same children in the same villages four years prior. It was also assumed that these villages experienced consistently or regularly high episodes of trachoma, based on previous studies.^41,42^ A larger randomly sampled survey may give more precise and more generalisable estimates of the prevalence of each sign, however if high TF prevalence is limited to these villages, a larger study area and population would decrease the prevalence estimates overall. Finally, no tests for infection were carried out in this study. This was because the guidance for these surveys^26^ suggested a purely clinical outcome with very little indication on how to interpret infection data from this age group.

This work is important for guiding the next steps for the PNG trachoma elimination programme. The predefined threshold for continuation of MDA was not met. However, cases of ocular *Ct* and all other signs of trachoma are present in PNG,^16,23^ albeit at a low prevalence, therefore trachoma is endemic and could potentially increase in prevalence in the future. It is important to implement surveillance systems to monitor this, and also maintain the capacity to manage incident cases of trichiasis (one of the WHO criteria for validation of trachoma elimination).^8^ WASH infrastructure improvements should continue to be made as part of a holistic, integrated approach to NTD management.^43^ These findings contribute to the wider discussion around the suitability of TF as a universal marker to guide MDA decisions and part-define elimination as a public health problem.^44^ There are a number of settings in which TF persists in the absence of a *Ct* infection signal^45–50^ and these are likely to increase as the global prevalence of trachoma decreases. Research into non-TF-based survey regimens is needed to better characterise programme options in the peri-elimination period.

## Conclusion

According to the predefined outcome criteria from this survey, MDA should not be conducted for trachoma elimination purposes in PNG. There are elements of the trachoma control programme, such as WASH access improvement, which would still be useful to prevent future increases in ocular *C. trachomatis* transmission and contribute towards integrated NTD control. Surveillance systems should be implemented to enable PNG to monitor potential future increases in trachoma prevalence. Given the limitations of TF as a marker in this and other settings, surveillance systems may benefit from use of non-TF markers.

## Figures and Tables

**Table 1:** Grading system used in this study.

Feature	Quantification	Grade
Pannus	<2.0 mm extension	0
(measured vertically from the upper limbus)^32^	2.0 to <4.0 mm extension	1
	4.0 to <6.0 mm extension	2
	≥6.0 mm extension	3
Herbert’s pits^32^	None	0
	One to three	1
	More than three, but not involving the entire upper lunular	2
	Entire upper lunular involved	3
	Cornea encircled or two rows of pits above	4
Cicatriciae^31^	None	0
	Fine scattered scars on the upper tarsal conjunctiva, or scars on other parts of the conjunctiva (mild)	1
	More severe scarring but without shortening or distortion of the upper tarsus (moderate)	2
	Scarring with distortion of the upper tarsus (severe)	3

**Table 2:** Pannus, Herbert’s pits and conjunctival scars in 14 selected villages of Papua New Guinea, 2020.

Sign	Severity	Number of participants positive (%)*
Scar	C0	1115 (95%)
	C1	54 (5%)
	C2	9 (1%)
	C3	0 (0%)
Pannus	0	1177 (>99%)
	1	1 (<1%)
	2 or 3	0 (0%)
Herbert’s pits	0	1177 (>99%)
	1	1 (<1%)
	2, 3 or 4	0 (0%)

* % rounded to nearest integer so may not add up to 100.

**Table 3:** Prevalence of scars with limbal signs in 14 selected villages of Papua New Guinea, 2020.

Conjunctival scar	Limbal signs			
	No	Yes	Primary outcomes	
			Any scar, any limbal sign	Severe scar, any limbal sign
C0	1114	1		
C1	54	0	**1 (0.08%)**	
C2	8	1		**1 (0.08%)**
C3	0	0		
